# The impact of the cumulative burden of LDL-c and hs-CRP on cardiovascular risk: a prospective, population-based study

**DOI:** 10.18632/aging.103365

**Published:** 2020-06-16

**Authors:** Jinglin Mo, Zimo Chen, Jie Xu, Anxin Wang, Xia Meng, Xingquan Zhao, Hao Li, Shouling Wu, Yongjun Wang

**Affiliations:** 1 Department of Neurology, Beijing Tiantan Hospital, Capital Medical University, Beijing, China; 2 China National Clinical Research Center for Neurological Diseases, Beijing, China; 3 Department of Cardiology, Kailuan General Hospital, Tangshan, China

**Keywords:** low density lipoprotein cholesterol, high-sensitivity C-reactive protein, cumulative burden, major adverse cardiac events

## Abstract

Background: This study aims to demonstrate the impact of the cumulative burden of low density lipoprotein-cholesterol (cumLDL-c) and high sensitivity C-reactive protein (cumhs-CRP) on cardiovascular risk.

Results: During the 4.62 (±0.70) years of follow-up, 2,148 (5.92%) participants had MACE. Both of cumLDL-c and cumhs-CRP were independent risk factors for MACE. In participants without cumLDL-c during 2006-2013, the participants with cumhs-CRP had higher MACE risk during the subsequent 5 years, than those without cumhs-CRP (hazard ratio [HR]: 1.24, 95% confidence interval [CI]:1.04-1.47). In addition, cumhs-CRP correlated with MACE in a cumhs-CRP level-dependent pattern.

Conclusion: This study validated the effects of residual inflammation risk in patients with low LDL-c Level in a general population, using long-term burdens of hs-CRP or LDL-c other than a single time-point level.

Method: The Kailuan study is a prospective, population-based study began in 2006. These total 36,421 participants completed 4 measurements of hs-CRP and LDL-c biennially from 2006-2013. Cumhs-CRP or cumLDL-c levels were calculated as the number of interval years multiplied by the Δhs-CRP (more than 2.0 mg/L) or ΔLDL-c (more than 2.6 mmol/L). Outcomes measured during follow-up (2012-2017) were defined as major adverse cardiac events (MACE), including ischemic stroke, myocardial infarction, and all-cause mortality.

## INTRODUCTION

Low density lipoprotein-cholesterol (LDL-c) is an independent risk factor for the development of atherosclerotic cardiovascular disease [1]. A number of randomized controlled trials have demonstrated the reduction in mortality and morbidity of cardiovascular disease (CVD) via primary [2–4] and secondary [4, 5] prevention through lipid-lowering therapy targeting LDL-c. However, although therapeutic targets for LDL-c can be reached, residual risk for CVD remains a challenge [6], possibly due to inflammation during atherosclerosis.

Previously, the CANTOS (Canakinumab anti-inflammatory Thrombosis Outcome Study) targeting residual inflammatory risk demonstrated that anti-inflammatory therapy significantly reduces the rate of recurrent cardiovascular events independent of decreases in lipid levels [7]. This is in agreement with the pathophysiological viewpoint that atherosclerosis is a disorder involving both hyperlipidemia and inflammation [8]. Moreover, a meta-analysis confirmed that both high sensitivity C-reactive protein (hs-CRP) and LDL-c levels similarly predict vascular risk [9]. Such evidence demonstrates that hs-CRP and LDL-c play important roles in primary and secondary prevention. However, most studies only demonstrated the harm of hs-CRP and LDL-c at single time-points without illustrating the effects of cumulative burdens. Moreover, it remains unclear whether the cumulative burden of hs-CRP may contribute to the risk of CVD in the presence of long-term low LDL-c.

To help address the above questions, we utilized a longitudinal cohort from the Kailuan Study to investigate the impact of cumulative hs-CRP burden on cardiovascular events without the presence of cumLDL-c.

## RESULTS

### General characteristics

**Figure 1:**
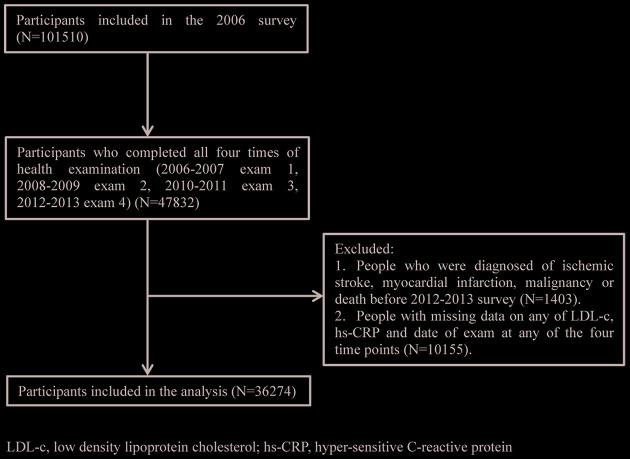
**Patients flow chart.**

Of the 101,510 participants enrolled in the Kailuan study, 47,828 patients completed all 4 examinations. A total of, 1,399 patients with ischemic stroke (IS), myocardial infarction (MI), malignancies, and/or death before examinations during 2012-2013 were excluded. Among the remaining 46,429 patients, 10,155 patients failed to undergo follow-up or examinations during 2006-2013, leaving 36,274 patients remaining for the final analysis, as shown in Figure 1.

From the final 36,274 patients, there were 2,148 (5.92%) major adverse cardiac events (MACE), including 864 (2.38%) IS, 224 (0.62%) MI, and 1,192 (3.29%) all-cause mortalities during follow-up over 4.62 (±0.70) years (2012-2017). Baseline characteristics stratified by cumulative burden of hs-CRP (cumhs-CRP) and cumulative burden of LDL-c (cumLDL-c) are summarized in Table 1. In general, participants with cumhs-CRP and cumLDL-c were more likely to be males, older, smokers, and non-drinkers. Moreover, these patients were more likely to have a history of diabetes mellitus, hypertension, hypercholesterolemia, and treatment with relevant medication. High levels of fast blood glucose (FBG), systolic blood pressure (SBP), diastolic blood pressure (DBP), triglyceride (TG), total cholesterol (TCHO), and lower high density lipoprotein cholesterol (HDL-c) were also found in this population.

**Table 1:** Baseline characteristics of participants as stratified by cumLDL-c and cumhs-CRP.

	**cumLDL = 0 cumCRP = 0**	**cumLDL > 0 cumCRP = 0**	**cumLDL = 0 cumCRP > 0**	**cumLDL> 0 cumCRP > 0**	***P* value**
Sample size	5229	11068	6456	13521	
Age (years), mean ± SD	45.8 (11.3)	46.9 (11.0)	50.2 (12.0)	50.5 (11.7)	<.0001
Body mass index, mean ± SD	24.1(3.1)	24.6 (3.0)	25.2 (3.4)	25.7 (3.3)	<.0001
Male, n (%)	3792 (72.5)	8536 (77.1)	4787 (74.2)	10087 (74.6)	<.0001
History of diabetes mellitus, n (%)	130 (2.5)	364 (3.3)	393 (6.1)	850 (6.3)	<.0001
History of hypertension, n (%)	561 (10.8)	1337 (12.2)	1124 (17.5)	2564 (19.1)	<.0001
History of hypercholesterolemia, n (%)	114 (2.2)	442 (4.0)	286 (4.5)	844 (6.3)	<.0001
History of antidiabetic medication, n (%)	77 (1.5)	242 (2.2)	273 (4.2)	607 (4.5)	<.0001
Antihypertensive medication, n (%)	340 (6.5)	862 (7.8)	831 (12.9)	1855 (13.7)	<.0001
Lipid-lowering medication, n (%)	15 (0.3)	75 (0.7)	65 (1.0)	163 (1.2)	<.0001
Previous or current smoker, n (%)	1427 (27.6)	3271 (29.6)	1809 (28.2)	4066 (30.1)	0.0008
Previous or current alcohol consumer, n (%)					<.0001
never	3876 (74.9)	7970 (72.4)	4815 (75.1)	9952 (74.2)	
past	8 (0.2)	6 (0.1)	17 (0.3)	29 (0.2)	
mild	1086 (21.0)	2505 (22.8)	1329 (20.7)	2828 (21.1)	
intermediate	188 (3.6)	477 (4.3)	236 (3.7)	538 (4.0)	
severe	19 (0.4)	47 (0.4)	16 (0.3)	61 (0.5)	
Physical exercise					<.0001
never	3069 (22.9)	1606 (25.1)	2345 (21.3)	1401 (27.1)	
sometimes	8845 (66.0)	4217 (65.8)	7505 (68.2)	3366 (65.0)	
usually	1490 (11.1)	588 (9.2)	1158 (10.5)	410 (7.9)	
Baseline systolic blood pressure, mmHg	126.6 (18.2)	129.2 (18.0)	131.7 (19.4)	134.0 (19.3)	<.0001
Baseline diastolic blood pressure, mmHg	81.6 (10.4)	83.0 (10.1)	83.2 (10.1)	84.3 (10.2)	<.0001
Fasting blood glucose, median (IQR), mmol/L	5.4 (1.5)	5.5 (2.0)	5.8 (1.9)	6.0 (2.0)	<.0001
Triglyceride level, median (IQR), mmol/L	1.0 (0.7-1.5)	1.2 (0.9-1.8)	1.2 (0.8-1.8)	1.4 (1.0-2.1)	<.0001
High density lipoprotein, median (IQR), mmol/L	1.4 (1.2-1.6)	1.4 (1.2-1.6)	1.3 (1.1-1.5)	1.3 (1.1-1.5)	<.0001
Total cholesterol, median (IQR), mmol/L	4.4 (4.0-4.8)	5.1 (4.5-5.7)	4.5 (4.0-5.1)	5.3 (4.8-6.0)	<.0001

Abbreviation: SD, standard deviation; IQR, interquartile range; cumLDL-c, cumulative burden of low density lipoprotein cholesterol; cumhs-CRP, cumulative burden of high sensitivity C-reactive protein; SBP, systolic blood pressure; DBP, diastolic blood pressure.

### Association between cumhs-CRP, cumLDL-c and clinical outcomes

**Figure 2:**
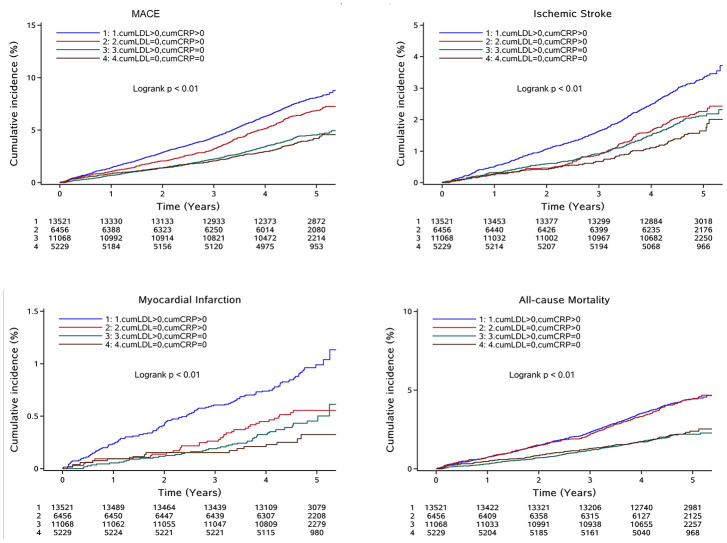
**Cumulative incidence of clinical outcomes in total participants stratified by cumLDL-c and cumhs-CRP;** Abbreviation: MACE: major adverse cardiac events; cumLDL-c, cumulative burden of low density lipoprotein cholesterol; cumhs-CRP, cumulative burden of high sensitivity C-reactive protein.

After multivariable adjustment for confounding factors including age, sex, smoking, alcohol consumption, SBP, FBG, HDL-c, and TG at baseline, compared with the reference group, the risk of clinical outcomes increased progressively with increased cumhs-CRP and cumLDL-c except for cumLDL-c in risk of all-cause mortality and cumhs-CRP in risk of ischemic stroke. (Table 2). In Table 3, participants were categorized into four groups: cumLDL-c = 0 and cumhs-CRP = 0, cumLDL-c = 0 and cumhs-CRP > 0, cumLDL-c > 0 and cumhs-CRP = 0, and cumLDL-c > 0 and cumhs-CRP > 0. The coexistence of cumLDL-c > 0 and cumhs-CRP > 0 was associated with greater MACE risk than that of the reference group (cumLDL-c = 0, cumhs-CRP = 0) across all adjusted models. Furthermore, significantly increased MACE risk was also observed in the cumLDL-c = 0, cumhs-CRP > 0 group, with a hazard ratio (HR) = 1.33, 95% confidence interval (CI)= 1.12-1.57. However, participants with cumLDL-c > 0 and cumhs-CRP = 0 did not show an increased risk of MACE (HR:1.03, 95%CI: 0.87-1.21). Same results were also observed in all-cause of mortality endpoint. The significantly increased IS and MI risk was only found in cumLDL-c > 0 and cumhs-CRP > 0 group when compared with reference group (cumLDL-c = 0, cumhs-CRP = 0) across all adjusted models. Cumulative incidence of clinical outcomes stratified by cumLDL-c and cumhs-CRP are presented by Kaplan-Meier survival curve analysis and the findings were consistent with the results analyzed by Cox regression models (Figure 2).

**Table 2:** Hazard ratios and 95% confidence intervals of MACE, ischemic stroke, myocardial infarction, and all-cause mortality, stratified by cumulative burden level of LDL-c, hs-CRP respectively.

	**MACE**			**Ischemic Stroke**			**Myocardial Infarction**			**All-Cause Mortality**		
	**Cases, n (person-year)**	**Incidence Rate (per 1000 person-year)**	**Hazard Ratio (95% CI)**	**Cases, n (person-year)**	**Incidence Rate (per 1000 person-year)**	**Hazard Ratio (95% CI)**	**Cases, n (person-year)**	**Incidence Rate (per 1000 person-year)**	**Hazard Ratio (95% CI)**	**Cases, n (person-year)**	**Incidence Rate (per 1000 person-year)**	**Hazard Ratio (95% CI)**
cumLDL-c												
cumLDL-c =0 (reference)	628 (54316)	11.8	1(ref.)	221 (55259)	4.0	1(ref.)	50 (55559)	0.9	1(ref.)	389 (54829)	3.33	1(ref.)
1	432 (38238)	12.0	1.07 (0.95-1.22)	193 (38857)	5.0	1.33 (1.09-1.61)	47 (39128)	1.2	1.35 (0.90-2.03)	219 (38727)	2.67	0.90 (0.76-1.07)
2	451 (37792)	12.5	1.03 (0.91-1.16)	189 (38392)	4.9	1.19 (0.98-1.45)	55 (38663)	1.4	1.56 (1.06-2.30)	237 (38277)	2.89	0.88 (0.75-1.04)
3	637 (36905)	17.6	1.27 (1.14-1.42)	261 (37791)	6.9	1.44 (1.20-1.73)	72 (38142)	1.9	1.85 (1.29-2.67)	347 (8196)	4.23	1.10 (0.95-1.27)
cumhs-CRP												
cumhs-CRP=0 (reference)	680 (75484)	9.29	1(ref.)	300 (76346)	3.93	1(ref.)	64 (76794)	0.83	1(ref.)	347 (76189)	4.55	1(ref.)
1	376 (30578)	12.82	1.20 (1.06-1.37)	155 (31072)	4.99	1.13 (0.93-1.38)	37 (31295)	1.18	1.24 (0.82-1.88)	213 (30944)	6.88	1.32 (1.12-1.57)
2	473 (30654)	16.21	1.35 (1.20-1.52)	198 (31376)	6.31	1.27 (1.05-1.52)	53 (31619)	1.68	1.57 (1.08-2.29)	248 (31176)	7.95	1.37 (1.16-1.62)
3	619 (30536)	20.66	1.55 (1.38-1.73)	211 (31505)	6.70	1.17 (0.98-1.41)	70 (31783)	2.20	1.93 (1.36-2.74)	384 (31130)	12.34	1.81 (1.56-2.11)

Adjusted for age, sex, smoker, alcohol intake, systolic blood pressure, fasting blood glucose, triglyceride, high density lipoprotein cholesterol at baseline.

Abbreviation: LDL-c, low density lipoprotein cholesterol; hs-CRP, high sensitivity C-reactive protein; MACE, major adverse cardiac events; cumLDL-c, cumulative burden of low density lipoprotein cholesterol; cumhs-CRP, cumulative burden of high sensitivity C-reactive protein.

**Table 3:** Hazard ratios and 95% confidence intervals of clinical outcomes stratified by cumulative burdens of LDL-c and hs-CRP.

**Outcomes**	**Cases, n (person-year)**	**Incidence Rate (per 1000 person-year)**	**Model 1HR(95%CI)**	**Model 2 HR(95%CI)**	**Model 3 HR(95%CI)**
MACE					
cumLDL-c>0 cumhs-CRP>0	1041 (61716)	16.87	1.62 (1.39-1.89)	1.64 (1.40-1.91)	1.48 (1.27-1.73)
cumLDL-c=0 cumhs-CRP>0	427 (30189)	14.14	1.33 (1.12-1.57)	1.33 (1.12-1.58)	1.24 (1.04-1.47)
cumLDL-c>0 cumhs-CRP=0	479 (51361)	9.33	1.07 (0.90-1.26)	1.07 (0.91-1.27)	1.03 (0.87-1.21)
cumLDL-c=0 cumhs-CRP=0	201 (24172)	8.32	Reference	Reference	Reference
Ischemic stroke					
cumLDL-c>0 cumhs-CRP>0	423 (63141)	6.70	1.70 (1.34-2.16)	1.67 (1.32-2.13)	1.45 (1.14-1.85)
cumLDL-c=0 cumhs-CRP>0	141 (30812)	4.58	1.15 (0.87-1.51)	1.11 (0.84-1.46)	1.01 (0.76-1.33)
cumLDL-c>0 cumhs-CRP=0	220 (51899)	4.24	1.23 (0.95-1.59)	1.21 (0.94-1.57)	1.14 (0.88-1.48)
cumLDL-c=0 cumhs-CRP=0	80 (24447)	3.27	Reference	Reference	Reference
Myocardial infarction					
cumLDL-c>0 cumhs-CRP>0	125 (63704)	1.96	2.66 (1.55-4.54)	2.61 (1.52-4.46)	2.47 (1.41-4.31)
cumLDL-c=0 cumhs-CRP>0	35 (30993)	1.13	1.50 (0.82-2.76)	1.49 (0.81-2.73)	1.48 (0.79-2.76)
cumLDL-c>0 cumhs-CRP=0	49 (52228)	0.94	1.46 (0.82-2.60)	1.45 (0.82-2.59)	1.50 (0.82-2.71)
cumLDL-c=0 cumhs-CRP=0	15 (24566)	0.61	Reference	Reference	Reference
All-cause of mortality
cumLDL-c>0 cumhs-CRP>0	570 (62774)	9.08	1.48 (1.21-1.81)	1.52 (1.24-1.87)	1.40 (1.14-1.72)
cumLDL-c=0 cumhs-CRP>0	275 (30476)	9.02	1.43 (1.15-1.78)	1.46 (1.17-1.83)	1.37 (1.10-1.72)
cumLDL-c>0 cumhs-CRP=0	233 (51835)	4.49	0.92 (0.73-1.15)	0.94 (0.75-1.17)	0.88 (0.70-1.11)
cumLDL-c=0 cumhs-CRP=0	114 (24354)	4.68	Reference	Reference	Reference

Abbreviation: MACE: major adverse cardiac events; cumLDL-c, cumulative burden of low density lipoprotein cholesterol; cumhs-CRP, cumulative burden of high sensitivity C-reactive protein; HR, hazard ratio; CI, confidence interval; Model 1 was adjusted for age and sex. Model 2 was adjusted for model 1 plus smoker, alcohol intake. Model 3 was adjusted for model 2 plus systolic blood pressure, fasting blood glucose, triglyceride, high density lipoprotein cholesterol at baseline.

### Risk of MACE in population with cumhs-CRP but without cumLDL-c

**Figure 3:**
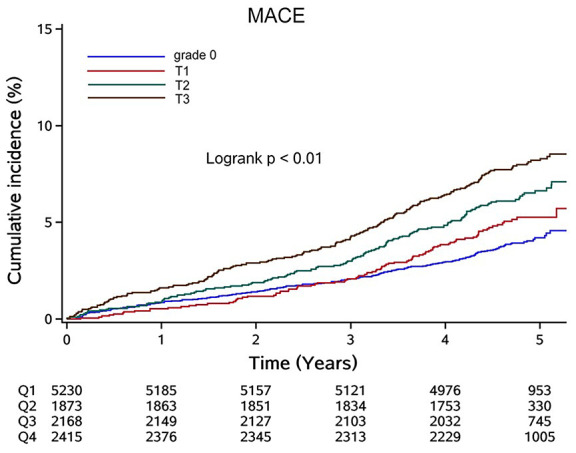
**Cumulative incidence of MACE in participants with cumLDL-c=0 stratified by cumhs-CRP level.** Abbreviation: MACE: major adverse cardiac events; cumLDL-c, cumulative burden of low density lipoprotein cholesterol; cumhs-CRP, cumulative burden of high sensitivity C-reactive protein.

We also conducted a sensitivity analysis of the population with cumLDL-c = 0. Figure 3 illustrates the Kaplan–Meier curves for MACE based on cumhs-CRP level. Participants with cumhs-CRP = 0 were designated grade 0 and the remaining participants designated either T1, T2, or T3 using cumhs-CRP tertiles. Although the overall incidence rate was low, there was a progressive increase in MACE rate with increasing cumhs-CRP tertile.

## DISCUSSION

Results from this large, community-based, prospective study involving repeated LDL-c and hs-CRP measurements emphasize the importance of detecting and controlling hs-CRP over long time periods, even if LDL-c levels are persistently low. The main findings of this study are as follows: 1) a large proportion (55.3%) of participants without cumLDL-c show detectable cumhs-CRP; 2) despite these low cumLDL-c levels, MACE risk remains high; and 3) cumhs-CRP levels correlate with MACE in a level-dependent manner.

We used 2 mg/L as the cutoff point for hs-CRP and 2.6 mmol/L as the cutoff point for LDL-c when calculating cumulative burden in our analyses. Although the European Society of Cardiology (ESC)/European Atherosclerosis Society (EAS) guidelines and American College of Cardiology (ACC)/American Heart Association (AHA) guidelines for lipid reduction propose 1.8 mmol/L as the lowest value for which lipid-lowering therapy is recommended for individuals for primary prevention [10, 11], a lower LDL-c (< 1.8 mmol/L) value was not associated with reduced risk of any clinical outcomes (coronary heart disease, stroke, and all-cause mortality) compared to the reference (≥ 1.8 mmol/L LDL-c) group [12]. Moreover, lower baseline LDL-c (< 1.8 mmol/L) is associated with a higher risk of intracranial hemorrhage [13]. We therefore propose that 2.6 mmol/L may be a more suitable for cut-off value for investigating cardiovascular risk in populations for primary prevention. In our study, the prevalence of cumhs-CRP> 0 was 55.1% in the total cohort and 55.8% in participants with cumLDL-c = 0. The proportions of patients with high hs-CRP were 43% and 47% in the PROVE-IT (Pravastatin or Atorvastatin Evaluation and Infection Therapy) trial [14] and IMPROVE-IT (Improved Reduction of Outcomes Vytorin Efficacy International Trial) trial [15], respectively.

Since atherosclerosis is a chronic, progressive disease that begins early in life and develops over the course of decades before clinical manifestation, single measurements of hs-CRP cannot estimate its cumulative effect on CVD risk. It is thus necessary to evaluate cumulative exposure and the related intensity and duration of risk factors. Previous studies mainly majored at the association between hs-CRP and MACE in population of secondary prevention, few studies focus on this issue in general population of primary prevention. Thus, it is necessary to evaluate cumulative exposure and the related intensity in general population. Our study determined the effect of the cumulative burden of hs-CRP on MACE in the presence of persistently low LDL-c using a large sample size. We took the advantage of 4 repeated measurements and found that even with persistently low LDL-c levels, a large proportion of participants with cumhs-CRP > 0 suffered from high MACE risk, with a cumhs-CRP level-dependent relationship with the level of MACE risk. This finding is in agreement with secondary analysis by CANTOS, which demonstrated that elevated hs-CRP is directly correlated to poorer clinical outcomes [16].

For primary prevention, the inflammatory biomarker hs-CRP was at least as valuable as LDL-c in predicting cardiovascular risk [9]. Hs-CRP independently predicts future cardiovascular events among healthy individuals [17, 18], as confirmed in more than 30 epidemiologic cohorts worldwide. In the Justification for the Use of statins in Prevention: an Intervention Trial Evaluating Rosuvastatin (JUPITER) trial, participants with LDL-c < 1.8 mmol/L and hs-CRP < 2 mg/L showed the strongest reductions in vascular events [19]. Similar results were also observed in IMPROVE-IT with ezetimibe/simvastatin [15] and the Further Cardiovascular Outcomes Research With PCSK9 Inhibition in Patients with Elevated Risk (FOURIER) trial with evolocumab in combination with optimized lipid-lowering therapy [20]. However, it remains unknown whether decreases in statin-mediated hs-CRP occurs by a mechanistic pathway that does not interfere with lipid levels when reducing the rates of cardiovascular events. CANTOS demonstrated that anti-inflammatory therapy with canakinumab is associated with a lower risk of recurrent cardiovascular events in patients with prior MI and increased baseline hs-CRP (≥2 mg/L), independent of lipid-lowering [21]. Due to the higher risk of MACE in population with cumhs-CRP > 0 and cumLDL-c = 0, our study favors the conclusion from CANTOS that inflammatory processes are important for the pathogenesis of CVD.

C-reactive protein is not only an inflammatory biomarker but also an important risk factor associated with ageing-related diseases including cardiovascular disease. The underlying biological mechanism linking cumhs-CRP and risk of CVD may involve the pro-atherogenic and pro-thrombotic role of inflammation in CVD risk. CRP may damage the endothelial glycocalyx, causing pro-atherogenic results [22]. In addition, tissue factors release from mononuclear, endothelial, and smooth muscle cells can be simulated by CRP [23–25], which would induce a pro-thrombotic state. Previous studies showed that older people who displayed an elevation in CRP levels over a decade experienced an increased risk of adverse aging outcomes [26]. Thus, it is necessary to take cumhs-CRP into consideration for risk evaluation especially for the elderly.

Multiple limitations must be considered for this study. First, this is an observational study, thus we are currently unable to generate causal conclusions. Second, due to the exclusion criteria, the inclusion of a number of participants with statin use from this community-based population was limited, therefore we could not conduct stratification analysis based on statin use, which is associated with both inflammation status and CVD risk [27]. Third, our study was based on an occupational cohort with unbalanced distributions of gender, social status, and economic status, among other demographic characteristics. These findings thus cannot be generalized directly to all populations.

In conclusion, this study validated the effects of residual inflammation risk in patients with low LDL-c Level in a general population, using long-term burdens of hs-CRP or LDL-c other than a single time-point level. This suggests that despite the role of cumLDL-c, cumhs-CRP should be also taken into consideration for risk evaluation.

## MATERIALS AND METHODS

### Study population

The Kailuan study was a prospective, community-based multicenter study carried out from June 2006 to October 2007 with follow-up surveys every 1-2 years. It collected information on adverse events experienced by the employees of the Kailuan Coal Group in order to investigate risk factors for chronic disease. Each participant underwent questionnaire assessments, physical examinations, and laboratory tests in a total number of 11 local hospitals responsible. Details on the design, objectives, recruitment, sampling, and quality-control have been previously published [28]. Participants who did not attend follow-up examinations or were diagnosed with malignancies, MI, or IS before 2012 were not eligible for the study.

This study was approved by the ethics committee of Kailuan General Hospital, Beijing Chaoyang Hospital, and Beijing Tiantan Hospital according to the principles expressed in the Declaration of Helsinki. Written informed consent was obtained from all participants. This study is registered in the International Clinical Trials Registry Platform (http://apps.who.int/trialsearch/Trial2.aspx?TrialID=ChiCTR-TNRC-11001489) with study ID: ChiCTR-TNRC-11001489.

### Laboratory measurements

Measurements of hs-CRP and LDL-c were performed biennially from during 2006-2007, 2008-2009, 2010-2011, and 2012-2013 to calculate cumulative burdens. Participant blood samples were collected after fasting for 8-12 h and transferred to vacuum tubes containing EDTA. All biochemical parameters were measured in the central laboratory of Kailuan General Hospital. LDL-c levels were measured by a direct testing method with an inter-assay coefficient of variation < 10% (Mind Bioengineering, Shanghai, China). Serum levels of hs-CRP were determined using a high-sensitivity nephelometry assay with a lower detection limit of 0.1 mg/L, an intra-assay coefficient of variation of 6.53%, and an inter-assay coefficient of variation of 4.78% (Cias Latex CRP-H, Kanto Chemical, Tokyo, Japan).

### Baseline data collection

Baseline data were collected via questionnaires and included information on demographics like age and sex, cigarette smoking status, alcohol intake, history of diabetes mellitus, hypertension and hypercholesterolemia status, and concomitant medications (e.g., antidiabetic, antihypertensive, lipid-lowering agents). Blood pressure was measured 2 times after participants were seated quietly for at least 5 min and blood pressure measurements averaged for analysis. Other biochemical parameters, including FBG, TG, HDL-c, and TCHO levels, were measured using an autoanalyzer (Hitachi 747, Hitachi) as described previously [29].

### The definition of cumulative burden of hs-CRP, LDL-c

Several studies calculated cumulative burden by multiplying mean values between 2 consecutive visits by years between visits [30, 31]. Since LDL-C and hs-CRP has been investigated for several years, they have the clear cut off value to define normal and abnormal value. Thus, we use cut-off value to modify the definition of cumulative burden in our study. The cumhs-CRP is defined as the weighted sum of the portion of 2 adjacent, averaged measurements that falls above the cutoff value that is then multiplied by time intervals between consecutive examinations in years:

Cumulative burden = [(value1+value2)/2 − cutoff ] × interval years_1-2_ + [(value2+value3)/2 − cutoff ] × interval years_2-3_ + [(value3+value4)/2 − cutoff ] × interval years_3-4_

The same formula was used for calculating the cumulative burden of LDL-c (cumLDL-c).

In our study, the cutoff values were defined as 2.0 mg/L and 2.6 mmol/L for cumhs-CRP and cumLDL-c, respectively. If the values of the cumulative burdens between 2 consecutive examinations were less than 0 this value would be considered as 0.

### Study outcomes and follow-up

Clinical outcomes were defined as MACE, including IS, MI. and all-cause mortality. Information regarding physician-diagnosed CVD events and all-cause mortality was collected using questionnaires during the biennial follow-ups from 2012 to 2017. The definition of ischemic stroke, according to ICD-10 criteria (codes I63 or I64), is based on characteristic signs, symptoms, and computed tomography scans or nuclear magnetic resonance images [32]. The definition of MI, according WHO Multinational Monitoring of Trends and Determinants in Cardiovascular Disease (MONICA) criteria, is based on the onset of angina pectoris, ischemic features in ECG, and a rise in cardiac serum markers [33]. The definition of all-cause mortality is death from any cause, confirmed by either a death certificate from the local citizen registry or the treating hospital. If clinical outcomes could not be certified with official documentation, reports during two consecutive follow-up periods by different proxies were considered acceptable. Follow-up appointments with the study population was continued from 2012-2017.

### Statistical analysis

Baseline characteristics were compared between groups categorized by cumhs-CRP and cumLDL-c levels. Continuous variables with skewed distributions are presented using medians (interquartile ranges [IQR]) and those with normal distribution are presented using the mean (standard deviation [SD]). Categorical variables are described using percentages (%). The incidence rates of the study outcomes were calculated by dividing the number of incident cases by person-years of the follow-up period. A multivariable-adjusted proportional-hazards model was used to investigate the association between cumLDL-c, cumhs-CRP, and the incidence of clinical outcome via multivariable Cox regression analyses. Model 1 was adjusted for age and sex. Model 2 was adjusted for age, sex, smoking, and alcohol consumption. Model 3 was adjusted for variables in Model 2 plus baseline SBP, FBG, HDL-c, and TG. Kaplan–Meier analyses were used to generate survival plots during the follow-up period and the significance of differences between groups was tested with the log-rank test.

A 2-sided P value of <0.05 was considered indicative of statistical significance. SAS software, version 9.4 (SAS Institute, Inc, Cary, NC), was used for all statistical analyses.
